# Single-cell RNA-seq reveals a key role for *Vibrio cholerae* Mak toxins in *Tetrahymena pyriformis* killing and bacterial survival

**DOI:** 10.3389/fmicb.2025.1729243

**Published:** 2026-01-22

**Authors:** Jonah M. Moon, M. Mozammel Hoque, Dana Ronin, Parisa Noorian, Joyce To, Scott A. Rice, Diane McDougald, Gustavo Espinoza-Vergara

**Affiliations:** 1 The Australian Institute for Microbiology and Infection, University of Technology Sydney, Sydney, NSW, Australia; 2 Section of Microbiology, The University of Copenhagen, Copenhagen, Denmark

**Keywords:** anti protozoal, protozoa, toxins, transcriptomics, *Vibrio cholerae*

## Abstract

In the environment, *Vibrio cholerae* employs multiple strategies to resist predation by heterotrophic protozoa. For example, *V. cholerae* biofilms release toxic compounds, such as ammonium and pyomelanin, which can kill protists, such as *Tetrahymena pyriformis. V. cholerae* has also been shown to survive intracellularly and can escape as viable cells inside protozoan-expelled food vacuoles (EFVs). We previously reported that *V. cholerae* encased in EFVs are hyperinfectious, establishing an important link between anti-protozoal strategies and bacterial virulence. Although the intracellular resistance and escape of *V. cholerae* in EFVs have been reported, the molecular mechanisms behind this remain poorly understood. Here, we used single-cell transcriptomics of *V. cholerae* exposed to *T. pyriformis* and captured a total of 5,344 bacterial cells with heterogeneous gene expression. Cells with the same pattern of gene expression were grouped, resulting in 11 clusters of cells with a unique gene expression profile. Genes encoding outer membrane proteins, F_1_F_0_-Na^+^/H^+^ ATPase, metabolites, and toxins showed differential expression among the clusters. Furthermore, the motility-associated killing factor (Mak) toxins were differentially expressed. The *V. cholerae* mutants Δ*makA,* Δ*makB,* and Δ*makE* were not capable of killing *T. pyriformis,* and Δ*makA and* Δ*makE* showed reduced survival inside EFVs compared to the wild type. These findings identify Mak toxins as key mediators of *V. cholerae* resistance to protozoan grazing and survival within EFVs. More broadly, our results provide mechanistic insight into grazing resistance, reveal factors facilitating persistence in EFVs, and underscore the interplay between environmental survival strategies and virulence in pathogenic bacteria.

## Introduction

*Vibrio cholerae* is a naturally occurring aquatic bacterium, of which only a small subset of strains causes cholera. In the environment, it interacts with many organisms, including heterotrophic (bacterivorous) protozoa. Bacterivorous protozoa, such as *Tetrahymena pyriformis*, feed on bacteria and package them in food vacuoles filled with digestive enzymes. However, pathogenic bacteria, such as *V. cholerae*, can resist protozoan grazing by the production of toxins, such as the PrtV protease (Vaitkevicius et al., 2006) and the release of toxic compounds, including pyomelanin (Noorian et al., 2017) and ammonia (Sun et al., 2015), which have been identified in *V. cholerae* biofilms.

Beyond these chemical defenses, *V. cholerae* produces motility-associated killing factor (Mak) proteins that form a tripartite complex, classified as pore-forming toxins (PFTs). Crystal structure analysis of these toxins revealed structural similarities to other PFTs, such as ClyA in *Escherichia coli* and NheA in *Bacillus cereus* (Nadeem et al., 2021a). Notably, while this family of PFTs is effective in killing mammalian macrophages, they are ineffective in *Acanthamoeba castellanii*, which displays innate resistance toward PFTs (Yabrag et al., 2025).

*V. cholerae* also resists intracellular digestion and subsequently escapes to the extracellular space inside protozoan expelled food vacuoles (EFVs) (Espinoza-Vergara et al., 2019). EFVs are packages of bacteria that resist the intracellular process and are released into the extracellular environment. This phenomenon has been shown in many pathogenic bacteria (Mueller et al., 1965). The inability to digest has been suggested as one of the drivers for EFV production, either by mechanisms utilized by the bacteria (Espinoza-Vergara et al., 2019), or by inefficiency in the protozoan digestion process (Thurman et al., 2010). In *V. cholerae,* we have identified that deletion of *ompU* reduces the number of EFVs formed by the protozoa. OmpU in *V. cholerae* has been reported to make the outer membrane less permeable (Wibbenmeyer et al., 2002), enhancing the protection of *V. cholerae* against multiple stressors, such as organic acids, antimicrobial peptides, reactive oxygen/nitrogen species, proteolytic enzymes, and low concentrations of essential metal ions, such as iron (Espinoza-Vergara et al., 2020). Thus, we have previously suggested that the expression of *ompU* inside the food vacuole allows *V. cholerae* to resist the digestion process, resulting in the egestion of *V. cholerae* in EFVs into the extracellular environment (Espinoza-Vergara et al., 2019).

Currently, two conditions are known to enhance *V. cholerae* infectivity in humans: (1) freshly shed bacteria from infected individuals and (2) biofilm-derived bacteria, which have an increased capacity to colonize the host (Nadeem et al., 2021a). More recently, the release of *V. cholerae* within EFVs has been identified as a third state of hyperinfectivity (Mitterer et al., 2020), significantly enhancing intestinal colonization.

Although EFV formation and release have been observed when some protozoa feed on a variety of pathogenic bacteria, the molecular mechanisms behind their release remain poorly understood. In *V. cholerae*, only the outer membrane porin, OmpU, has been shown to be involved in the process of EFV formation (Espinoza-Vergara et al., 2019).

When implementing transcriptomics to elucidate molecular mechanisms in bacteria, one of the main limitations is capturing gene expression at a single-cell level. It is known that within bacterial communities, there are subpopulations of cells that display heterogeneous gene expression profiles to account for the survival of the whole community (Eldar and Elowitz, 2010), a process termed “stochastic” gene expression (Kuchina et al., 2021). The detection of the specific gene expression profiles of these subpopulations cannot be achieved using the current bulk transcriptomic approaches. This becomes more challenging when the goal is to determine the expression of bacterial genes in different microenvironments, such as those observed in bacteria–eukaryotic cell co-incubations.

To gain insights into the general grazing resistance mechanisms of *V. cholerae* against *T. pyriformis*, here, we used MicroSPLiT (Kuchina et al., 2021), a novel single-cell transcriptomic approach to elucidate gene expression at a single-cell level, with the capacity to capture distinctive gene expression profiles in very small and rare subpopulations (up to 0.1% of the whole community). Using this tool, we identified several genes with differential expression across 11 subpopulations of cells with a distinctive gene expression profile. We demonstrate that the newly identified pore-forming toxin (Nadeem et al., 2021a), codified by the *makABE* genes, is involved in the killing of *T. pyriformis* and in the survival of *V. cholerae* in EFVs. These findings increase our understanding of the molecular mechanisms displayed by *V. cholerae* for predation resistance, which contributes to improving our understanding of the dissemination of this relevant pathogen.

## Results

### Single-cell RNA-seq reveals heterogeneous gene expression of *Vibrio cholerae* in the presence of *Tetrahymena pyriformis*

**Figure 1:**
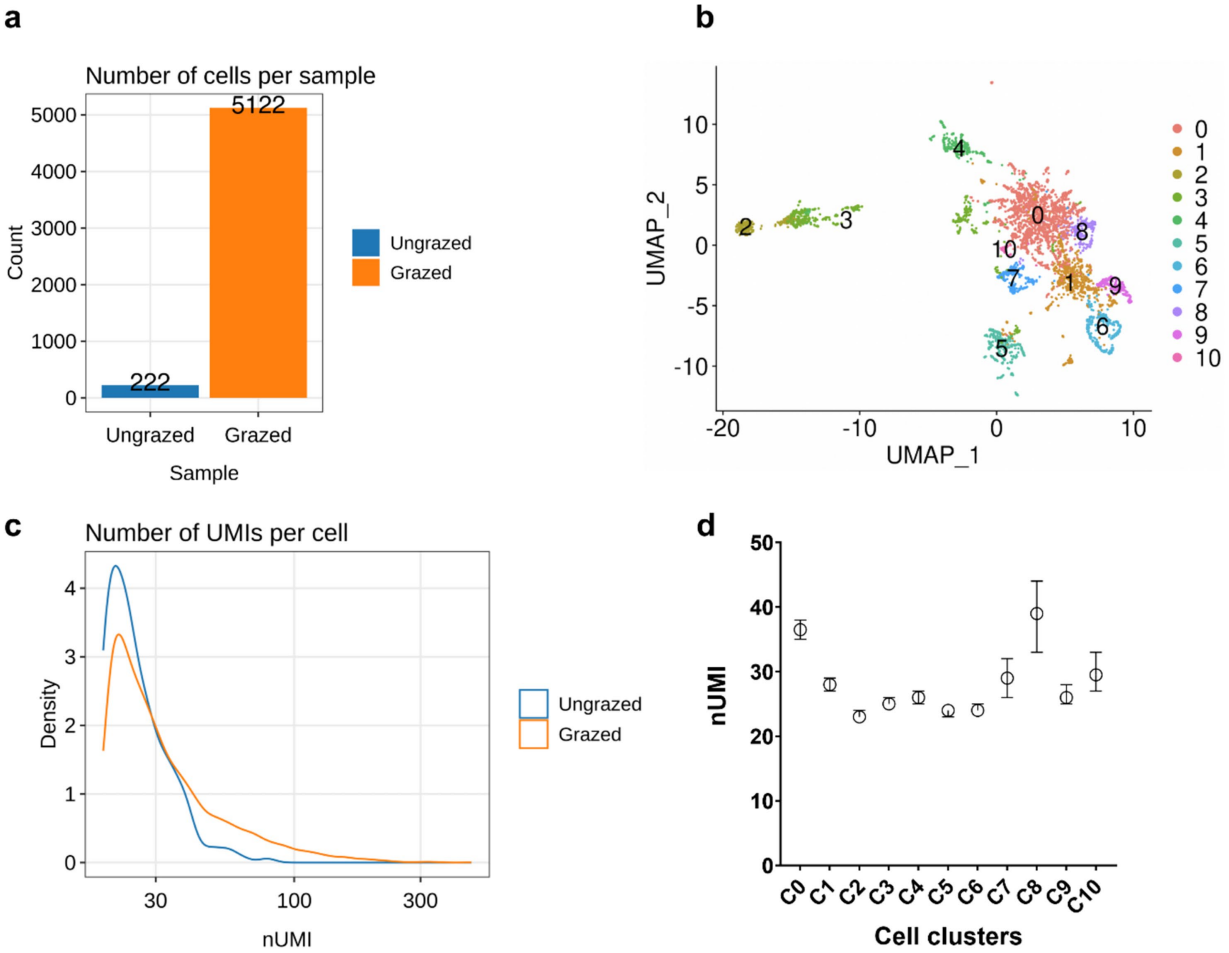
Single-cell RNA-seq of *V. cholerae* reveals heterogeneous gene expression in the presence of *T. pyriformis*. (**a)** Number of cells captured in the ungrazed (control) and grazed (experimental) conditions. (**b)** Uniform manifold approximation and projection (UMAP) of two-dimensional plots of the 11 clusters of cells displaying differential gene expression within the grazed population. (**c)** Number of mRNA UMI counts per cell. (**d)** Number of mRNA UMI per cluster of gene expression. The results are shown as median with 95% CI.

To elucidate the molecular mechanisms involved in the grazing resistance of *V. cholerae* to *T. pyriformis*, we applied MicroSPLiT (Kuchina et al., 2021), a single-cell transcriptomic approach for bacteria. We co-incubated *V. cholerae* and *T. pyriformis* in artificial seawater (ASW) with no addition of nutrients. A total of 5,344 bacteria were individually barcoded, with 222 cells identified under control conditions [ungrazed, starved cells incubated in ASW at room temperature (RT) for 6 h], and 5,122 cells identified in the experimental condition (grazed, cells incubated in ASW in the presence of *T. pyriformis* at RT for 6 h; Figure 1a).

Using the standard workflow of the R package seurat on the single-cell expression matrices, we resolved unique transcriptional states by clustering cells with a similar gene expression pattern, obtaining a total of 11 (0–10) clusters of cells with a distinctive gene expression profile (Figure 1b). The median number of Unique Molecular Identifiers (UMI) that are directly related to the number of mRNA transcripts per cell was low (Figure 1c) compared to other reports of bacterial single cell transcriptomics (average median for UMI/cell is >200; Thurman et al., 2010). Low UMI may be due to poor cell permeabilization. Therefore, permeabilization of the *V. cholerae* cells used to prepare the single-cell RNA library was confirmed using Ovalbumin Alexa Fluor 488 conjugate (Thermofisher, Waltham, Massachusetts, USA), resulting in the accumulation of green-fluorescent signal inside the cells. To further confirm that the low UMI/cell observed is a limitation of the experiment and not a barcoding inefficiency of cellular RNA, we compared the number of UMIs observed among the different clusters. The median UMI between clusters was significantly different (Figure 1d), indicating that even under low-nutrient conditions, where the overall number of UMI counts is low, the observed variation reflects genuine differences in metabolic activity across the cell clusters.

### Differential gene expression observed in the 11 clusters

As observed in previous single-cell RNA-seq reports in bacteria, clusters of gene expression can be related to specific biological processes. For example, cluster 0 represents 33.54% (1718/5122) of all grazed cells in this experiment and reveals a slight upregulation of *ompU* (log_2_FC = 0.58, adjusted*P*value = <1×10^−6^), a gene that we have previously found to be important for EFV production (Espinoza-Vergara et al., 2019). Cluster 7 reveals upregulation of flagellar genes (*flhA*, *flhF*, *flaD,* and *flaC*, log_2_FC > 5.5, adjusted *p-*value = <1×10^−6^) and represents 3.68% (189/5122) of the total grazed population. The presence of specific subpopulations of cells overexpressing flagellar genes has been observed in previous barcoding-based single-cell RNA-seq reports (McNulty et al., 2023).

**Figure 2:**
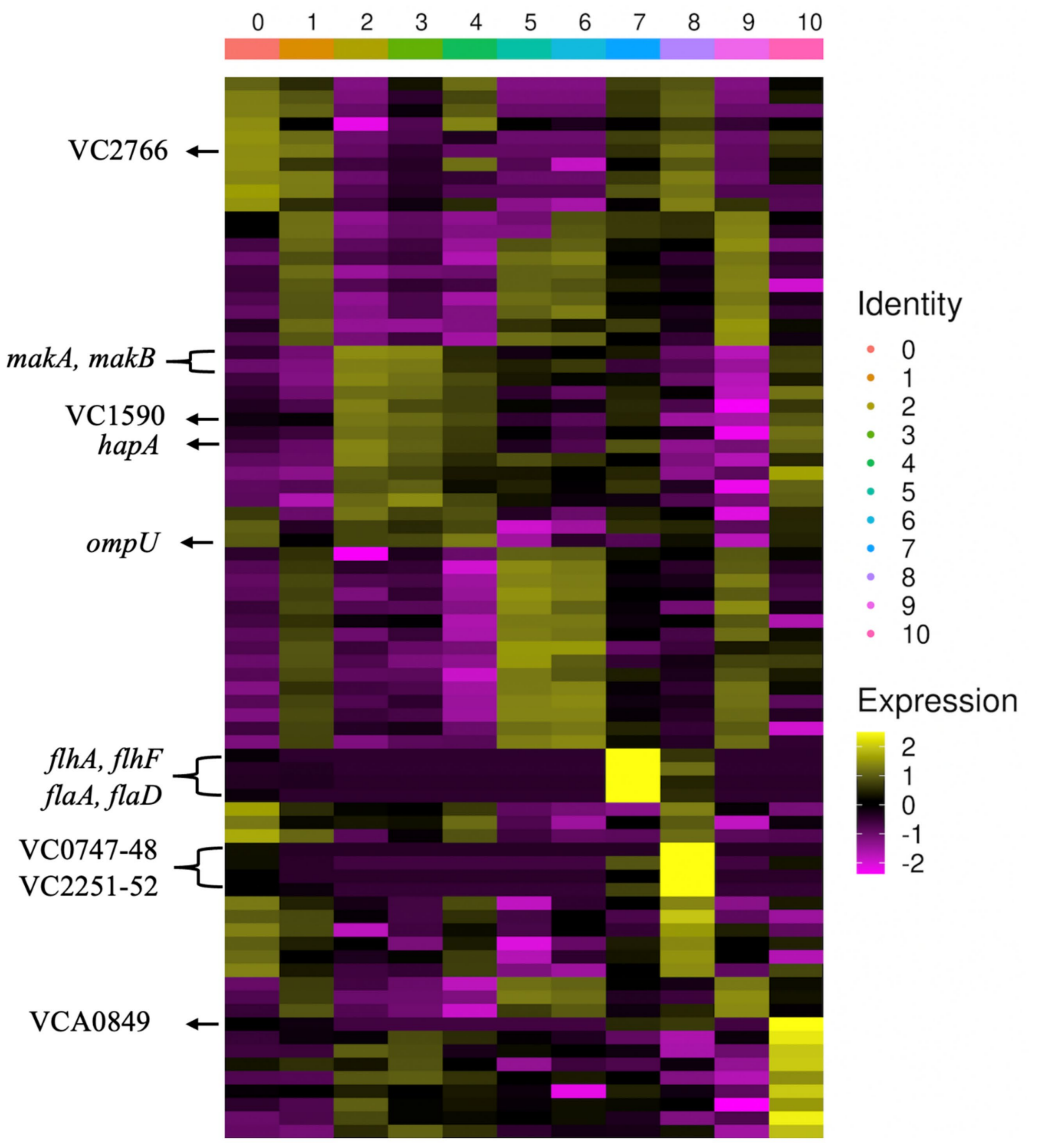
Heatmap of gene expression (*z*-score of log-transformed values) from 5,344 *V. cholerae* cells organized into 11 clusters (0–10). Upregulation of genes is indicated by yellow, and downregulation is indicated by magenta. On the left side, there is a selection of genes indicated.

Genes with significant differential expression were identified among the different clusters (Figure 2; Supplementary File S1). Clusters 1, 5, 6, and 9 show upregulation of genes related to tRNAs and ribosomal proteins. In addition to the upregulation of *ompU*, cluster 0 shows upregulation of genes involved in the synthesis of the F_1_F_0_ -ATPase (VC2764, VC2766, and VC2770, avg. log_2_FC > 1.4, adjusted *p-*value = <1×10^−6^), which is related to ATP synthesis (von Ballmoos et al., 2009) and for survival of acidic conditions by pathogenic bacteria (Sun, 2016).

Cluster 8 cells displayed upregulation of the transcriptional factor *icsR* (VC0747, log_2_FC = 5.5, adjusted *p-*value = <1×10^−6^), a global transcriptional regulator which is involved in iron homeostasis and stress response (Lim and Choi, 2014), and the cysteine desulfurase gene (VC0748, log_2_FC = 5.7, adjusted *p-*value = <1×10^−6^), which is important for mobilization of sulfur for iron–sulfur clusters involved in iron homeostasis (Zheng and Dean, 1994). VC2251 and VC2252, *ompH,* which is involved in the chaperoning of outer membrane proteins, and the outer membrane protein assembly factor BamA (Mathieu-Denoncourt and Duperthuy, 2025), respectively, are also upregulated (log_2_FC > 5.9, adjusted *p-*value = <1×10^−6^). Cluster 10 shows strong upregulation of *craA* (VCA0849, log_2_FC = 7.2, adjusted *p-*value = <1×10^−6^), a gene positively regulated by c-di-GMP, suggesting that this cluster identifies cells producing c-di-GMP, a key intracellular signal in *V. cholerae* involved in the positive regulation of biofilm genes (Beck et al., 2021). Cyclic AMP receptor protein (CRP, VC2614) is also shown to be upregulated in this cluster (log_2_FC = 2.8, adjusted *p-*value = 2.9×10^−2^), and it has previously been demonstrated that HapR and CRP can bind to shared DNA sites, while also maintaining the ability to directly interact with each other, primarily in the blocking of the RNA polymerase binding site of CRP by HapR (Walker et al., 2023).

Interestingly, one of the most differentially expressed genes in the various clusters is a hypothetical 65-amino acid protein, VC0713. We observed that the expression of this peptide positively correlates with the expression of *ompU* in the different clusters, suggesting a potential connection in the expression of these two genes.

### Heterogeneous expression of toxins appears to be a key anti-protozoal strategy in *Vibrio cholerae*

**Figure 3:**
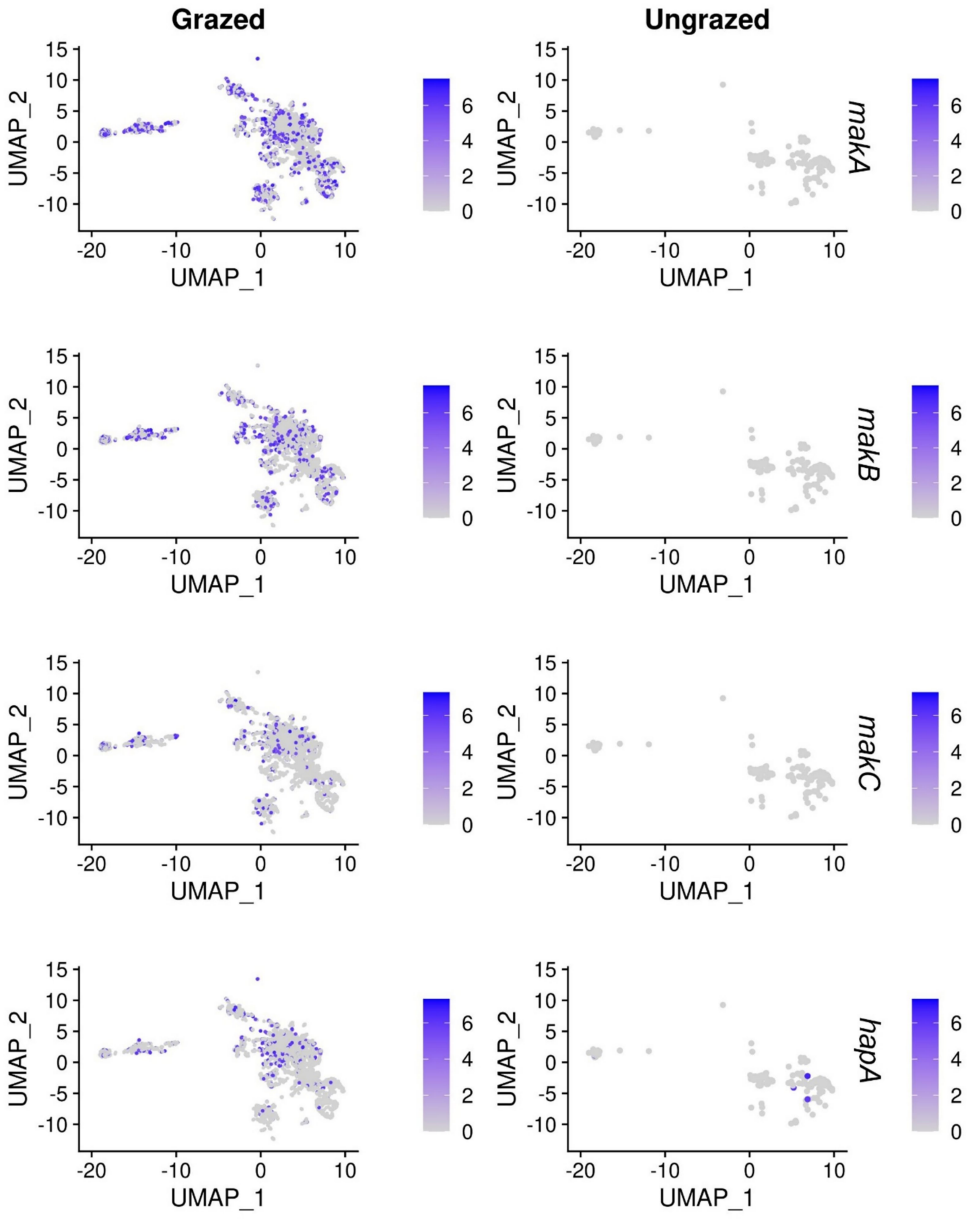
Differential expression of HapR-regulated toxins. Single-cell gene expressions of *makA*, *makB*, *makC,* and *hapA* (VCA0865) are highlighted in the UMAP plots. The left column corresponds to the expression in the grazing condition, while the right column shows the ungrazed condition (control).

The expression of virulence factors has been demonstrated to be heterogeneous in bacterial populations (Davis, 2020; Gonzalez and Mavridou, 2019). In these experiments, we observed that there is differential expression of toxins that are regulated by the master regulator of quorum sensing in *V. cholerae*, HapR. Upregulation of the motility-associated killing (*mak*) factor genes *makA*, *makB,* and *makC* (log_2_FC > 1.2, adjusted *p-*value < 2×10^−18^), along with the hemagglutinin (HA)/protease gene (VCA0865), *hapA* (log_2_FC = 1.47, adjusted *p-*value = 1.1×10^−13^), was observed under grazing pressure (Figure 3). Interestingly, clusters 2 and 3 that show the strongest upregulation of the toxins, also show upregulation of VC1590 (acetolactate synthase) and VC1591 (oxidoreductase) (log_2_FC > 1.5, adjusted *p-*values < 2×10^−15^), which are involved in the production of 2,3 butanediol. It has recently been shown in *Clostridium perfringens* that acetate is involved in the regulation of the secreted pore-forming toxin NetB (McNulty et al., 2023). This could suggest that in *V. cholerae*, the production of Mak toxins could also be regulated by a metabolite-toxin-regulated system, as was shown in *C. perfringens*.

### Mak toxins are required by *Vibrio cholerae* for killing of *Tetrahymena pyriformis* and survival in EFVs

**Figure 4:**
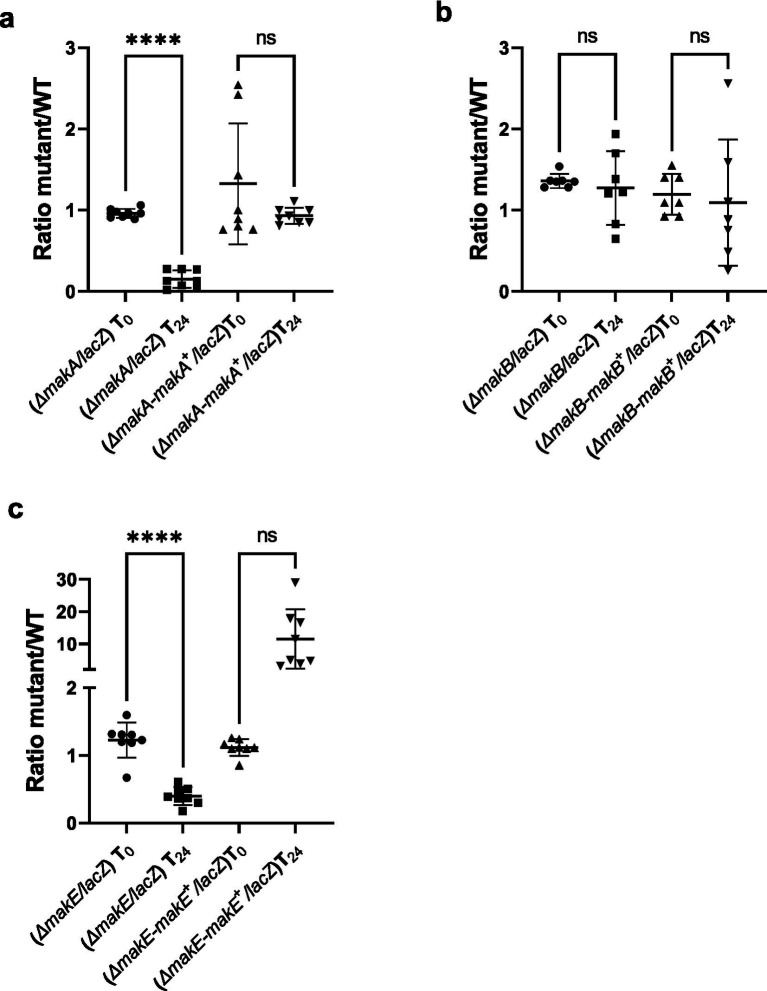
Importance of Mak toxins in *V. cholerae* for survival within EFVs. All complemented mutants described here are notated as Δ*mak𝑥-mak*𝑥^+^, where 𝑥 is the specific gene. **(a)** Δ*makA*; **(b)** Δ*makB*; **(c)** Δ*makE*. Bacterial survival in EFVs was calculated as the number of colonies of Δ*mak* strains divided by the number of colonies of WT after 24 h incubation of *V. cholerae* and *T. pyriformis* in ASW. Co-incubation of *V. cholerae* WT (Δ*lacZ*) or Δ*mak* strains was independently performed with *T. pyriformis* at an infectious dose of 10,000. The initial inoculum of both strains was mixed 50:50 and plated on X-gal LB plates at 30 °C to assess blue (mutant) and white (WT) number of colonies (T_0_). After overnight incubation, equal amount of EFVs produced independently with the WT and Δ*mak* strains were mixed and digested with Triton-X100 to release the bacterial cells inside. The mixture was then plated on X-gal LB plates at 30 °C to assess blue (mutant) and white (WT) number of colonies (T_24_). Data are from three independent biological replicates and are shown as the average. Significant differences were determined using one-way ANOVA with Tukey’s multiple comparisons test. ****p < 0.0001.

**Figure 5:**
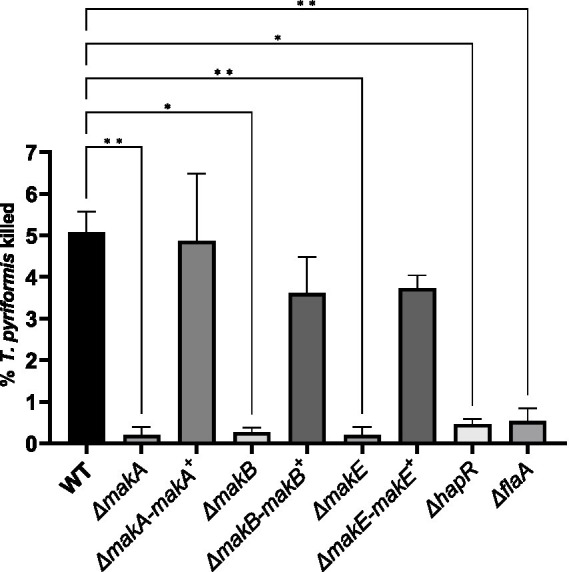
Percentage of *T. pyriformis* cells killed by *V. cholerae*. All complemented mutants described here are notated as Δ*mak𝑥-mak𝑥*^+^ where *x*is the specific gene. WT, Δ*mak* strains, Δ*hapR*, and Δ*flaA* were incubated with *T. pyriformis* for ~5 h of co-incubation in ASW at RT. The percentage of killing was assessed by counting the number of dead cells at the bottom of the well, divided by the total number of cells, multiplied by 100. Data are from three independent biological replicates and are shown as the average ± s.d. Significant differences were determined using an unpaired *t*-test with Welch’s correction (*p < 0.05, **p < 0.01).

To investigate and validate the role of Mak toxins in the grazing defense of *V. cholerae* against *T. pyriformis*, we constructed an in-frame Δ*mak*A, Δ*makB,* and Δ*makE* deletion mutant. After 24-h incubation, the *makA* and *makE* mutants show a survival defect (16 and 31% survival compared to WT, respectively) in the EFVs. However, the survival is restored to WT levels after complementing the *makA* gene *in trans* in the Δ*mak*A strain (Figure 4a). In the *makE* mutant, complementation resulted in enhanced EFV survival when compared to the WT (Figure 4c). Additionally, it was observed that deletion and complementation of the *makB* mutant had no effect on bacterial survival (Figure 4b). Furthermore, after approximately 5 h of co-incubation, we observed that the WT strain kills a fraction of the *T. pyriformis* population (5.07%), and this killing effect is not observed in the Δ*makA,* Δ*makB*, or Δ*makE* deletions. Complementation of these three genes *in trans* fully restores the killing effect in the Δ*mak*A, Δ*makB,* and Δ*makE* strains (4.87, 3.60, and 3.73%, respectively) (Figure 5; Supplementary Figures 1–2; Supplementary Videos 1–3). Additionally, *ΔhapR* and *ΔflaA* mutants were tested, and the *mak* mutants were unable to kill the protozoa. HapR is the regulator of the mak operon (Dongre et al., 2018), while FlaA is required for flagellum formation, the site through which the Mak tripartite toxin is secreted (Nadeem et al., 2021a). Together, these data suggest that *makA* and *makE* in *V. cholerae* are involved in intracellular survival, and all three parts of the mak toxin are required for the killing of *T. pyriformis*.

To investigate the potential importance of other genes identified in the single-cell transcriptomic data that are involved in bacterial survival, mutants were generated and incubated for 24 h. After 24 h, the only mutant that was shown to have a differential survival rate compared to the WT was Δ*nlpD* (Supplementary Figure S3).

## Discussion

Our results show that gene expression heterogeneity is observed in the interaction of *V. cholerae* and *T. pyriformis*, and several genes were identified that were differentially expressed. Although the data revealed low numbers of UMIs per cell, which is directly related to the low number of mRNA transcripts, significant differential expression of genes was observed. We conclude that the low number of transcripts per cell was not due to poor cell permeabilization; rather, the data suggest that it could be a technical limitation for the capture of mRNA from cells incubated in low nutrient conditions, as has been observed in single-cell transcriptomics of Gram-negative bacteria during the stationary phase of growth (Blattman et al., 2020).

Although *Vibrio* concentrations reported in environmental waters are often on the order of ~10^3^ cells mL^−1^ (Batabyal et al., 2016; Neogi et al., 2012), our experiments were conducted at 1 × 10^8^ bacterial cells mL^−1^ with 1 × 10^3^ *T. pyriformis* cells mL^−1^, consistent with our prior work (Espinoza-Vergara et al., 2019; Hoque et al., 2023; Rahman et al., 2022). We used this higher bacterial density to achieve a sufficiently high multiplicity of infection (MOI) to reproducibly capture phenotype-mediated effects on *T. pyriformis* health and on bacterial survival over the experimental time frame. Practically, lower bacterial numbers led to sporadic encounters between predators and bacteria, making the relevant phenotypes difficult to detect and quantify with confidence. Furthermore, the use of >5 × 10^7^ bacterial cells was also dictated by the downstream single-cell workflow, as this input is the minimum required for robust MicroSPLiT scRNA-seq library preparation.

The data reported here confirms our previous observation that OmpU plays an important role in resistance to predation by *T. pyriformis* (Espinoza-Vergara et al., 2019), as we observed upregulation of the *ompU* gene in a large number of cells. In the same group (cluster 0), genes encoding the F_1_F_0_-Na^+^/H^+^ ATPase were also upregulated. This suggests that these cells encounter an acidic environment, such as the one inside the food vacuole, since it has been reported that the presence and activation of the F_1_F_0_-Na^+^/H^+^ ATPase system is a signature of acidic tolerance in other pathogenic bacteria (Sun, 2016).

One of the most differentially expressed operons was the Mak operon, which is positively regulated by HapR (Dongre et al., 2018), the master regulator of quorum sensing in *V. cholerae*. HapR is activated at high cell densities, whereas at low cell densities, *hapR* is not expressed, leading to biofilm formation (Teschler et al., 2015). Our results show that the Mak operon contributes to grazing resistance, suggesting that Mak-expressing cells are not engaged in biofilm formation. This may also explain the absence of pathways associated with pyomelanin or ammonium production, compounds released by *V. cholerae* biofilms (Noorian et al., 2017; Sun et al., 2015).

Specifically, our work revealed that Mak toxins are involved in the killing of *T. pyriformis* as well as in the survival of *V. cholerae* in EFVs. MakA is a concentration-dependent pore/tubule-forming toxin, active under mildly acidic conditions (pH < 6.5) (Jia et al., 2022; Nadeem et al., 2022; Herrera et al., 2022). When paired with MakB and MakE, it forms a tripartite pore-forming complex, MakABE, which can cause lysis of mammalian cells (Nadeem et al., 2021a; Herrera et al., 2022). In addition, MakA can function to recruit cholesterol and plasma membranes into mammalian phagosomes at low concentrations (Corkery et al., 2021; Nadeem et al., 2021b). These observations together suggest that Mak toxins may be produced outside the protozoan host when *V. cholerae* populations are high. Following ingestion of *V. cholerae* by the protozoan, the toxins could enter the host and, within the acidified food vacuoles of *T. pyriformis*, form pores in the membrane of the food vacuole. This disruption would enable *V. cholerae* to escape into the cytoplasm, ultimately leading to host cell lysis. *V. cholerae* might survive by recruitment of plasma membrane components, including sterols, a fact that could explain the presence of membranous aggregates that we have previously observed when *V. cholerae* is contained inside food vacuoles within *T. pyriformis* and EFVs (Espinoza-Vergara et al., 2019).

Previous studies have shown that Mak toxin production is heterogeneous and depends on the protozoan host. In *A. castellanii*, Mak toxins are downregulated, indicating that production of these PFTs is host specific (Hoque et al., 2023). Additionally, *A. castellanii* is resistant to PFTs (Yabrag et al., 2025) and supports intracellular growth of *V. cholerae*, which can establish an endosymbiotic relationship within the amoebal cytoplasm (Abd et al., 2005; Abd et al., 2007). Together, these facts suggest that the expression of Mak toxins in *V. cholerae* is host-dependent.

In relation to the anti-grazing activity of Mak toxins, we show that all three Mak proteins are required for killing *T. pyriformis*, indicating that the MakABE complex is essential for this effect. However, when examining bacterial survival in EFVs, our results indicate that only MakA and MakE are required, whereas deletion of *makB* has no effect. The reason for this discrepancy remains unclear but may reflect structural and functional differences among the three Mak proteins. As shown previously, only MakA and MakE bind host membranes (Nadeem et al., 2021a). If survival within EFVs depends on Mak-mediated interactions with host membranes, the inability of MakB to bind these membranes could explain its non-essential role in this context.

Increasing evidence has elucidated the mechanism of action of Mak toxins in animal models and mammalian cell lines (Jia et al., 2022; Nadeem et al., 2022; Herrera et al., 2022; Corkery et al., 2021; Nadeem et al., 2021b). However, evidence directly linking Mak toxins to the pathogenesis of *V. cholerae* in these models remains limited. Our findings demonstrate that Mak toxins serve as a key anti-grazing mechanism in an environmental niche for *V. cholerae*. Previous studies have shown that the Mak toxin genomic island is present in over 60% of *V. cholerae* genomes in public databases (Herrera et al., 2022). Given that *V. cholerae* spends most of its life cycle in the environment, these results strongly support the idea that Mak toxins play an important role in its long-term environmental persistence. This provides a more direct functional role for Mak toxins, explaining the persistence of *V. cholerae* as an adaptive survival strategy and a defense mechanism against protozoan predation.

Our results demonstrate the use of this technology for identifying molecular factors in bacteria within complex environments, such as those found in bacteria–eukaryote interactions. We show that Mak toxins enhance the fitness of *V. cholerae* when contained within EFVs, a recognized transmission vector for infectious diseases. The combined presence of all three toxins can subsequently kill the predatory cell, offering a previously unrecognized explanation for the persistence of this genomic island in *V. cholerae*.

## Methods

### Strains and growth conditions

Organisms used in this study are listed in Supplementary Table S1. Bacterial strains were routinely grown in lysogeny broth (LB) and on LB agar plates. *V. cholerae* mutants were constructed by splicing by overlap extension PCR (Horton et al., 1989), and natural transformation was performed on chitin flakes (Dalia et al., 2014). Complementation was done using the expression vector pBAD24. Bacteria carrying the expression vector were grown in LB broth at 37 °C containing ampicillin 100 μg mL^−1^ and 0.2% arabinose for gene expression.

*Tetrahymena pyriformis* was routinely passaged in 10 mL of growth medium containing peptone-yeast-glucose (PYG) (20 g l^−1^ proteose peptone, 1 g l^−1^ yeast extract) supplemented with 0.1 × M9 minimal medium (6 g l^−1^ NaH_2_PO_4_, 3 g l^−1^ K_2_PO_4_, 0.5 g l^−1^ NaCl, 1 g l^−1^ NH_4_Cl) and 0.1-M sterile-filtered glucose in 25 cm^2^ tissue culture flasks with ventilated caps (Sarstedt Inc., Nümbrecht, Germany) and incubated statically at room temperature (RT).

Prior to experiments, 250 μL of *T. pyriformis* were passaged in 10 mL of ASW medium (5.6 g l^−1^ NaCl, 0.470 g l^−1^ Na_2_SO_4_, 0.026 g l^−1^ NaHCO_3_, 0.08 g l^−1^ KCl, 0.013 g l^−1^ KBr, 0.600 g l^−1^ MgCl_2_.6H_2_O, 0.130 g l^−1^ CaCl_2_.2H_2_O, 0.002 g l^−1^ SrCl_2_.6H_2_O and 0.002 g l^−1^ H_3_BO_3_) supplemented with 1% heat-killed *Pseudomonas aeruginosa* PAO1 (HKB) in a 25 cm^2^ tissue culture flask, and further incubated at RT statically for 24 h before enumeration and use. This process is necessary to remove the nutrient media and to acclimatize the ciliate to phagotrophic feeding (Noorian et al., 2017; Espinoza-Vergara et al., 2019).

To prepare heat-killed bacteria (HKB), *P. aeruginosa* was grown overnight in LB at 37 °C with shaking at 200 rpm and adjusted to (OD_600_ = 1.0; 10^9^ cells mL^−1^) in ASW. The tubes were then transferred to a water bath at 65 °C for 2 h and then tested for viability by plating on LB agar plates at 37 °C for 2 days. HKB stocks were stored at −20 °C.

### Co-incubation of *Vibrio cholerae* and *Tetrahymena pyriformis*

*V. cholerae* A1552 was co-incubated with *T. pyriformis* in ASW. Briefly, *T. pyriformis* were enumerated by microscopy and adjusted to 10^3^ cells mL^−1^ and added to co-cultures of *V. cholerae* A1552 adjusted to 10^8^ cells mL^−1^ in ASW using a spectrophotometer (OD_600_ nm). Co-incubation was performed for 5 h at RT, 60 rpm. After incubation, TritonX-100 was added at a final concentration of 1% to release intracellular and EFV-encased bacteria. The cells were centrifuged for 5 min (3,220 × *g*) at 4 °C, and the cell pellet was resuspended in a freshly made 4% ice-cold paraformaldehyde solution. Fixed cells were incubated overnight at 4 °C.

### Cell permeabilization

Fixed cells were centrifuged (5,500 × *g*) for 10 min. All centrifugation steps were performed at 5500 × *g*, 4 °C, 10 min. After centrifugation, cells were washed with 1 mL of 0.1 M Tris–HCl pH7 (Sigma) and centrifuged again. Cells were then resuspended in 250 μL of cold-PBS (pH 7.4, Sigma) supplemented with RNAase inhibitors (Enzymatics RI and Superase-IN) as indicated by the MicroSPLiT protocol (Kuchina et al., 2021). Following resuspension, 250 μL of 100% cold ethanol was added and incubated for 1 min at RT. Cells were again centrifuged and resuspended in a solution containing 0.04% Tween20 and 50 mM EDTA. The suspension was incubated for 3 min on ice. Following incubation, 750 μL of cold PBS supplemented with RNAase inhibitors was added. After centrifugation, cells were resuspended in 100 μL of a lysozyme buffer (0.1 M Tris pH7, 50 mM EDTA, 1 mg mL^−1^ lysozyme) and incubated at RT for 15 min. Permeabilization was assessed by mixing 50 μL of permeabilized cells with Ovalbumin Alexa Fluor 488 conjugate (Thermofisher, Waltham, Massachusetts, USA), following the manufacturer’s recommendations.

### *In situ* cDNA barcoding and NGS library preparation

*In situ* polyA tailing, reverse transcription, ligation barcoding, and library preparation were performed following the steps published in the MicroSPLiT protocol (Kuchina et al., 2021).

### Data processing

Single-cell gene expression matrices were obtained by mapping the cDNA reads (Read 1) to the reference genome of *V. cholerae* O1 El Tor strain N16961 using the STAR aligner (v2.7.10b) with the addition of several STARsolo parameters (−-genomeDir, −-readFilesIn, −-soloType, −-soloCBposition, −-soloUMIposition, −-soloCBwhitelist, −-soloCBmatchWLtype, −-soloUMIdedup, −-soloFeatures, −-soloMultiMappers) (Dobin et al., 2013). The resulting matrices were analyzed using the standard workflow of the R package seurat (v4.3.0) with default parameters except otherwise indicated (Butler et al., 2018). The data were log-transformed using the ‘NormalizeData(normalization.method = ‘LogNormalize’, scale.factor = 10,000)’ function. Five hundred most variable genes were selected using ‘FindVariableFeatures(selection.method = ‘vst’, nfeatures = 500)’ and z-scored using ‘ScaleData’. Linear dimensional reduction was performed using principal component analysis (PCA). Louvain clustering algorithm was used to generate clusters using the function ‘FindNeighbors(dims = 1:10)’ and ‘FindClusters(resolution = 0.5)’. Uniform manifold approximation and projection (UMAP) non-linear dimensional reduction techniques were used to visualize and explore the datasets using ‘RunUMAP(dims = 1:10)’ function. Differential markers in each cluster were identified using the ‘FindAllMarkers(logfc.threshold = 0.2)’ function. The average expression of the top 10 differentially expressed markers in each cluster was used to generate a heatmap using the ‘DoHeatMap’ function. The FeaturePlot function was used to visualize specific marker expression in the clustered population.

### Killing of *Tetrahymena pyriformis*

To assess the number of dead *T. pyriformis*, co-incubations were performed as described before. Briefly, *V. cholerae* and *T. pyriformis* were co-incubated in ASW for 5 h at RT. After approximately 5 h, dead cells accumulated at the bottom of the well and were counted using an inverted epifluorescence microscope (Nikon Eclipse Ti inverted microscope).

### Survival of *Vibrio cholerae* in EFVs

To produce EFVs, *V. cholerae* strains, Δ*lacZ* (WT) and mutants, were co-incubated with *T. pyriformis* independently in ASW. Briefly, *T. pyriformis* were enumerated by microscopy and adjusted to 10^3^ cells mL^−1^ and added to co-cultures of *V. cholerae*, adjusted to 10^8^ cells mL^−1^ in ASW using a spectrophotometer (OD_600_ nm). After overnight incubation at RT, samples were analyzed using an inverted epifluorescence microscope (Nikon Eclipse Ti inverted microscope) to detect the presence of EFVs in the supernatant. To purify *V. cholerae*-EFVs, supernatants were filtered (by gravity) several times through 8-μm filters (Millipore, Darmstadt, Germany), and the filters containing EFVs were suspended in 1 mL ASW. The EFVs were incubated for 1 h with gentamicin 300 μg mL^−1^ at RT to kill any remaining extracellular bacteria. After gentamicin treatment, *V. cholerae*-EFVs pellets were collected by centrifugation (3,220 × *g* for 20 min), washed three times in ASW, and suspended in 1 mL of ASW. Finally, Triton X-100 was added to a final concentration of 1% to lyse the EFVs and release bacterial cells. The lysed EFV aliquots (WT and mutant) were mixed 1:1 and plated on LB supplemented with X-Gal (60 μg mL^−1^) to differentiate between the *V. cholerae* Δ*lacZ* (white colonies) and the mutant strains (blue colonies).

### Total survival assay

*Vibrio cholerae* WT, Δ*nlpD,* Δ*VC1590,* Δ*VCA1097,* and Δ*hapA* strains were co-incubated with *T. pyriformis* independently in ASW. *T. pyriformis* were enumerated by microscopy and adjusted to 10^3^ cells mL^−1^ and added to co-cultures of *V. cholerae* adjusted to 10^8^ cells mL^−1^ in ASW using a spectrophotometer (OD_600_ nm). After 24 h, samples were treated with Triton X-100 to a final concentration of 1% to lyse the EFVs and release bacterial cells from the protozoa. These aliquots were plated onto LB and enumerated for survival.

### Data analysis

Statistical analysis was performed using GraphPad Prism version 8.4.3 for Windows, GraphPad Software, La Jolla, California, United States.^1^ Data that did not follow a Gaussian distribution was determined by analyzing the frequency distribution through the Shapiro–Wilk normality test. Two-tailed Student’s *t*-tests were used to compare means between experimental samples and controls using Welch’s *t*-test correction. For experiments including multiple samples, one-way ANOVA was used for the analysis, the Kruskal-Wallis test was used to compare the medians of non-normally distributed data, and Tukey’s test was used to compare the averages of normally distributed data.

## Data Availability

The datasets presented in this study can be found online in the NCBI GEO (https://www.ncbi.nlm.nih.gov/geo), under the accession code GSE307158.

## Footnotes

1
www.graphpad.com
