# Supplementary material for: Single-cell RNA-seq reveals a key role for Vibrio cholerae Mak toxins in Tetrahymena pyriformis killing and bacterial survival
Source: Front Microbiol. 2026 Jan 22;16:1729243. doi: 10.3389/fmicb.2025.1729243 (PMC12872930; doi:10.3389/fmicb.2025.1729243)
Supplement: Supplementary file 1 [file Data_Sheet_1.pdf]

Supplementary information:

Single-cell RNA-seq reveals a key role for *Vibrio cholerae* Mak toxins in *Tetrahymena pyriformis* killing and bacterial survival

Jonah M. Moon[1], M. Mozammel Hoque[1], Dana Ronin[2], Parisa Noorian[1], Joyce To[1], Scott A. Rice[1], Diane McDougald[1], Gustavo Espinoza-Vergara[1*].

[1] The Australian Institute for Microbiology & Infection, University of Technology Sydney, Sydney, Ultimo NSW 2007, Australia.

[2]Section of Microbiology, The University of Copenhagen, Copenhagen, Denmark

*Correspondence to: Gustavo.EspinozaVergara@uts.edu.au

Contents:

Supplementary Table 1

Supplementary Table 2

Supplementary Figure 1

Supplementary Figure 2

Supplementary Figure 3

Supplementary Figure 4

Supplementary Figure 5

Supplementary File Legends

Supplementary Video Legends

Data Availability

Supplementary Table 1 - List of bacterial and protozoan strains and plasmids

**Strain and plasmids**	**Properties**	**Origin**
V. cholerae A1552 S	Wild type, O1, El Tor, Inaba, Smooth, Rif^r	Laboratory collection
V. cholerae A1552 S Δ*lac*Z	O1, El Tor, Inaba, Smooth, Rif^r Δ*lac*Z	Laboratory collection
V. cholerae A1552 S *ΔmakA*	O1, El Tor, Inaba, Smooth, Rif^r *ΔmakA*	This study
V. cholerae ΔmakA-makA$^+$	*V. cholerae* A1552 S *ΔmakA* with makA complemented *in trans* in pBAD24.	This study
V. cholerae A1552 S *ΔmakB*	O1, El Tor, Inaba, Smooth, Rif^r *ΔmakB*	This study
V. cholerae ΔmakB-makB$^+$	*V. cholerae* A1552 S *ΔmakB* with makB complemented *in trans* in pBAD24.	This study
V. cholerae A1552 S *ΔmakE*	O1, El Tor, Inaba, Smooth, Rif^r *ΔmakE*	This study
V. cholerae ΔmakE-makE$^+$	*V. cholerae* A1552 S *ΔmakE* with makE complemented *in trans* in pBAD24.	This study
V. cholerae A1552 S *ΔnlpD*	O1, El Tor, Inaba, Smooth, Rif^r *ΔnlpD*	This study

V. cholerae A1552 S *ΔVC1590*	O1, El Tor, Inaba, Smooth, Rif^r *ΔVC1590*	This study
V. cholerae A1552 S *ΔVCA1097*	O1, El Tor, Inaba, Smooth, Rif^r *ΔVCA1097*	This study
V. cholerae A1552 S *ΔhapA*	O1, El Tor, Inaba, Smooth, Rif^r *ΔhapA*	This study
V. cholerae A1552 S *ΔhapR*	O1, El Tor, Inaba, Smooth, Rif^r *ΔhapR*	Laboratory collection
V. cholerae A1552 S *ΔflaA*	O1, El Tor, Inaba, Smooth, Rif^r *ΔflaA*	Laboratory collection
Escherichia coli BW20767	RP42tet::Mu1kan::Tn7integrant uidA(λλMlu1)::pir+ recA1 creB510 leu63 hsdR17 endA1 zbf5 thi	ATCC 47084
pBAD24	Cloning vector with arabinose inducible promoter, Amp^r	Laboratory collection
**Protozoan strains**		
T. pyriformis	Wild type	ATCC 205063

Supplementary Table 2 – Statistical comparison tests of Fig. 1d 2 –

Dunn's multiple comparisons test	Mean rank diff.	Significant?	Summary	Adjusted P Value
C0 vs. C1	730.2	Yes	****	<0.0001
C0 vs. C2	1517	Yes	****	<0.0001
C0 vs. C3	1098	Yes	****	<0.0001
C0 vs. C4	957.3	Yes	****	<0.0001
C0 vs. C5	1447	Yes	****	<0.0001
C0 vs. C6	1277	Yes	****	<0.0001
C0 vs. C7	574.2	Yes	****	<0.0001
C0 vs. C8	-84	No	ns	>0.9999
C0 vs. C9	925	Yes	****	<0.0001
C0 vs. C10	540.9	No	ns	0.5871
C1 vs. C2	786.6	Yes	****	<0.0001
C1 vs. C3	367.6	Yes	***	0.0009
C1 vs. C4	227.1	No	ns	0.8014
C1 vs. C5	716.6	Yes	****	<0.0001
C1 vs. C6	547.2	Yes	****	<0.0001
C1 vs. C7	-156	No	ns	>0.9999
C1 vs. C8	-814.2	Yes	****	<0.0001
C1 vs. C9	194.8	No	ns	>0.9999
C1 vs. C10	-189.2	No	ns	>0.9999
C2 vs. C3	-419	Yes	***	0.0003
C2 vs. C4	-559.5	Yes	****	<0.0001
C2 vs. C5	-69.97	No	ns	>0.9999
C2 vs. C6	-239.4	No	ns	>0.9999
C2 vs. C7	-942.5	Yes	****	<0.0001
C2 vs. C8	-1601	Yes	****	<0.0001
C2 vs. C9	-591.7	Yes	**	0.0038
C2 vs. C10	-975.8	Yes	***	0.0005
C3 vs. C4	-140.5	No	ns	>0.9999
C3 vs. C5	349	Yes	*	0.0267
C3 vs. C6	179.6	No	ns	>0.9999
C3 vs. C7	-523.6	Yes	**	0.0016
C3 vs. C8	-1182	Yes	****	<0.0001
C3 vs. C9	-172.7	No	ns	>0.9999
C3 vs. C10	-556.8	No	ns	0.5972
C4 vs. C5	489.5	Yes	***	0.0002
C4 vs. C6	320.1	No	ns	0.218
C4 vs. C7	-383.1	No	ns	0.1861
C4 vs. C8	-1041	Yes	****	<0.0001
C4 vs. C9	-32.27	No	ns	>0.9999
C4 vs. C10	-416.3	No	ns	>0.9999
C5 vs. C6	-169.4	No	ns	>0.9999
C5 vs. C7	-872.6	Yes	****	<0.0001
C5 vs. C8	-1531	Yes	****	<0.0001
C5 vs. C9	-521.8	Yes	*	0.0388
C5 vs. C10	-905.8	Yes	**	0.0026
C6 vs. C7	-703.2	Yes	****	<0.0001

Supplementary Table 2 – Statistical comparison tests of Fig. 1d 2 –

C6 vs. C8	-1361	Yes	****	<0.0001
C6 vs. C9	-352.4	No	ns	>0.9999
C6 vs. C10	-736.4	No	ns	0.0571
C7 vs. C8	-658.2	Yes	***	0.0004
C7 vs. C9	350.8	No	ns	>0.9999
C7 vs. C10	-33.25	No	ns	>0.9999
C8 vs. C9	1009	Yes	****	<0.0001
C8 vs. C10	624.9	No	ns	0.3869
C9 vs. C10	-384.1	No	ns	>0.9999

Supplementary Figure 1

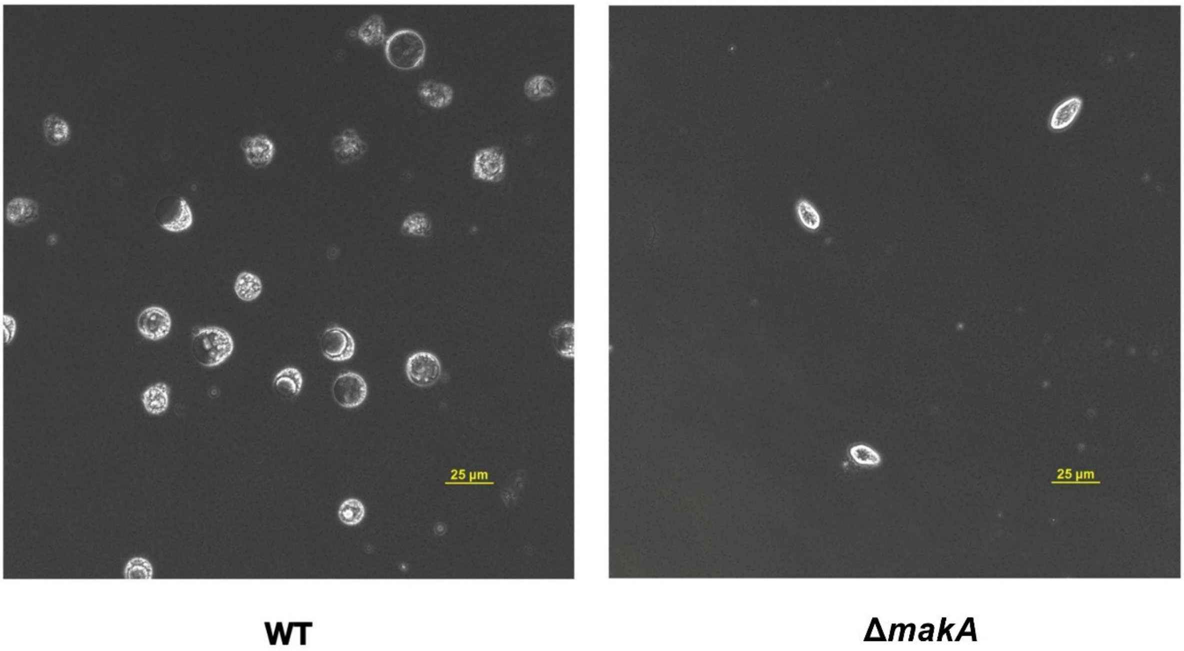

Supplementary Figure 1. Killing effect of *V. cholerae* against *T. pyriformis*. The left side shows many dead *T. pyriformis* cells concentrated at the bottom of the well. On the right side, the co-incubation of *V. cholerae* Δ*makA* mutant with *T. pyriformis* shows no dead *Tetrahymena* cells. Scale bar, 25 µm. Images are representative of three independent experiments.

Supplementary Figure 2

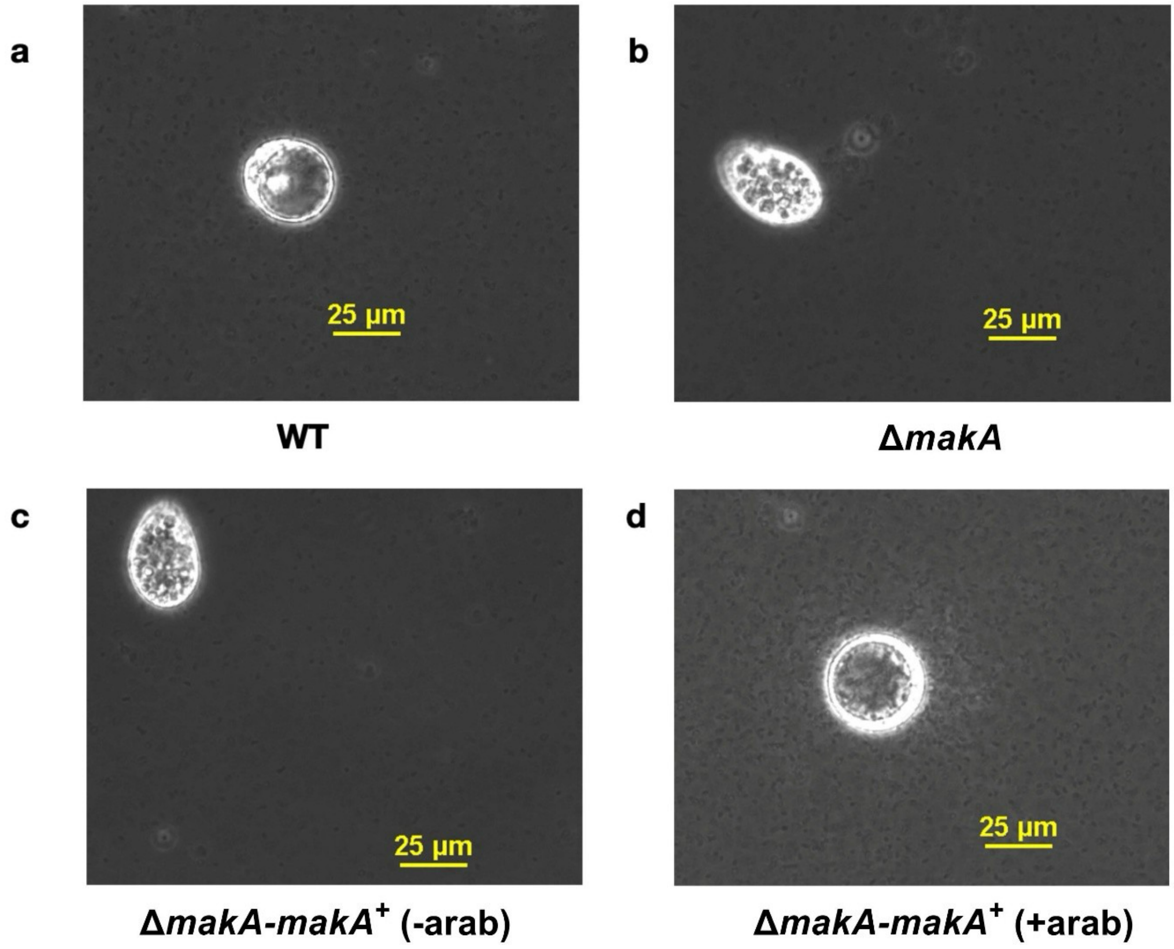

Supplementary Figure 2. Complementation of the *makA* gene *in trans* in the *V. cholerae* Δ*makA* restores the killing effect against *T. pyriformis*. a. WT strain killing *T. pyriformis*, an enlarged intracellular vesicle is observed. b, c. The killing effect against *T. pyriformis* is not observed in either the Δ*makA* strain (b) or in the Δ*makA*-*makA*$^{+}$ with no addition of arabinose (c). d. The killing effect of *T. pyriformis* is restored in the Δ*makA*-*makA*$^{+}$ in the presence of arabinose. Scale bar, 25 µm. Images are representative of three independent experiments.

Supplementary Figure 3

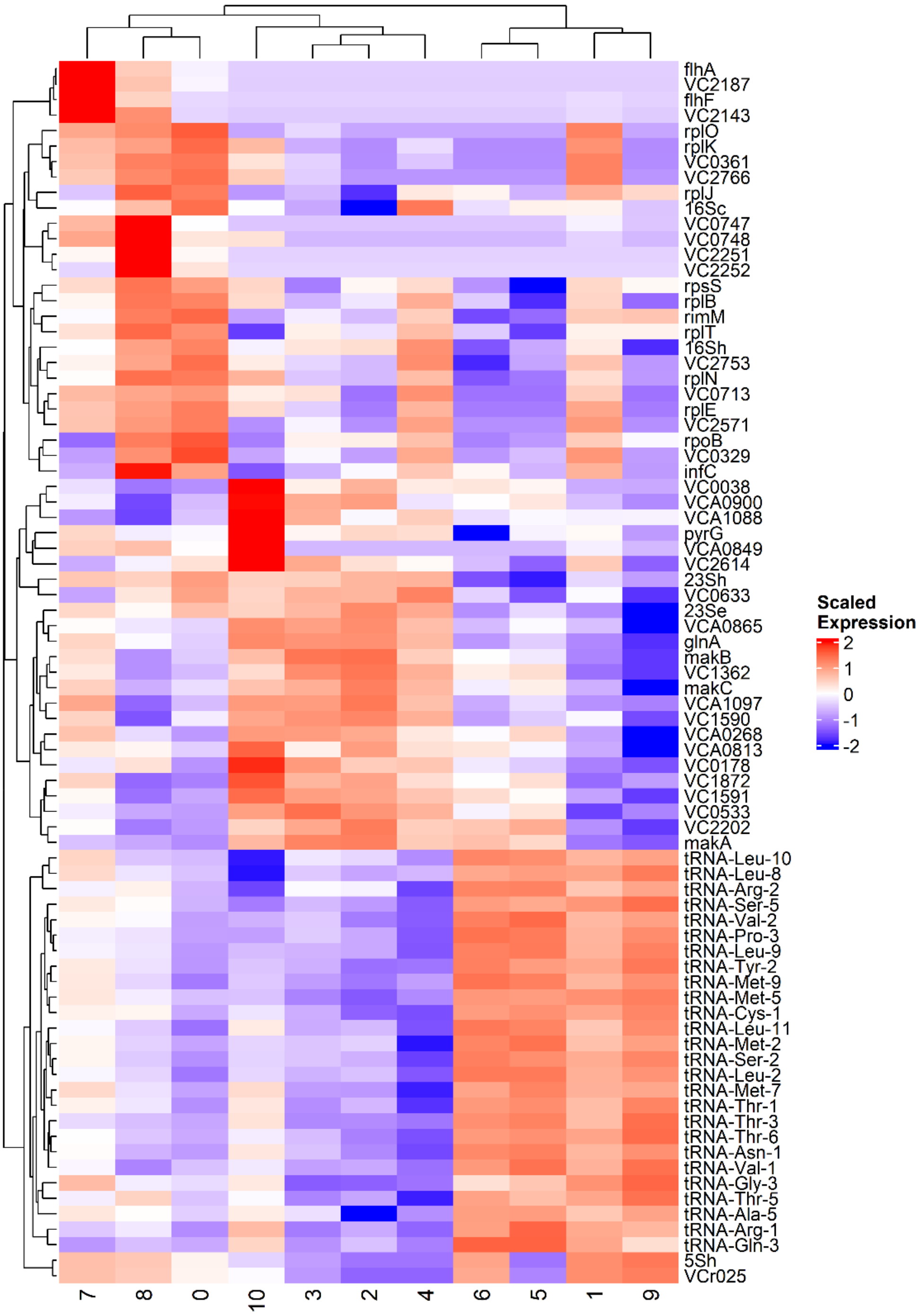

Supplementary Figure 3. A full presentation of the data presented in Fig. 2, displaying a heatmap of gene expression (*z*-score of log transformed values) from 5,344 *V. cholerae* cells organized into eleven clusters (0 to 10).

Supplementary Figure 4

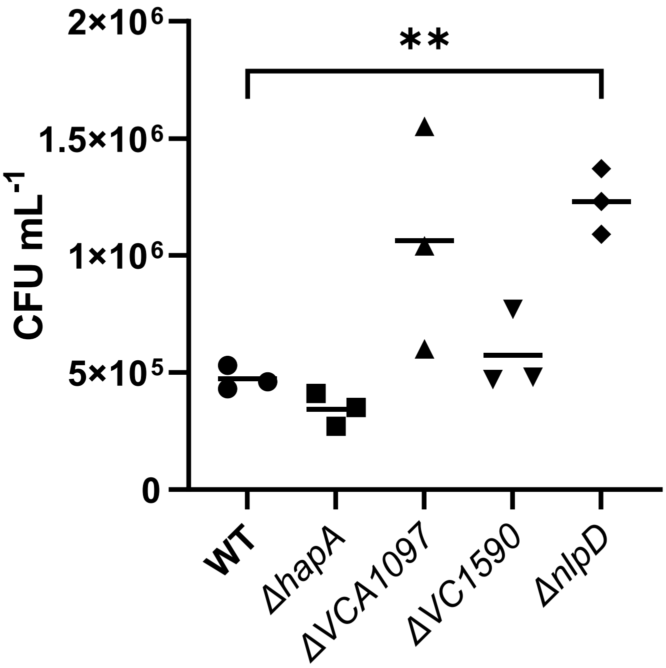

Supplementary Figure 4. Total bacterial survival of different *V. cholerae* mutants in the presence of *T. pyriformis*. Candidate gene for deletions were selected based on their significant upregulation in the single-cell transcriptomic dataset and were subsequently used to validate the transcriptomic data. Bacterial survival was performed by independently incubating *V. cholerae* and *T. pyriformis* for 24 h in ASW at an infectious dose of 10,000. After the incubation time was completed strains were mixed and digested with Triton-X100 to release the bacterial cells inside EFVs or inside of *T. pyriformis*. This was then plated onto LB agar at 30°C to assess survival. Data are from three independent biological replicates and are shown as the average. Significant differences were determined using one-way ANOVA with Tukey's multiple comparisons test. $**P < 0.01$.

Supplementary Figure 5

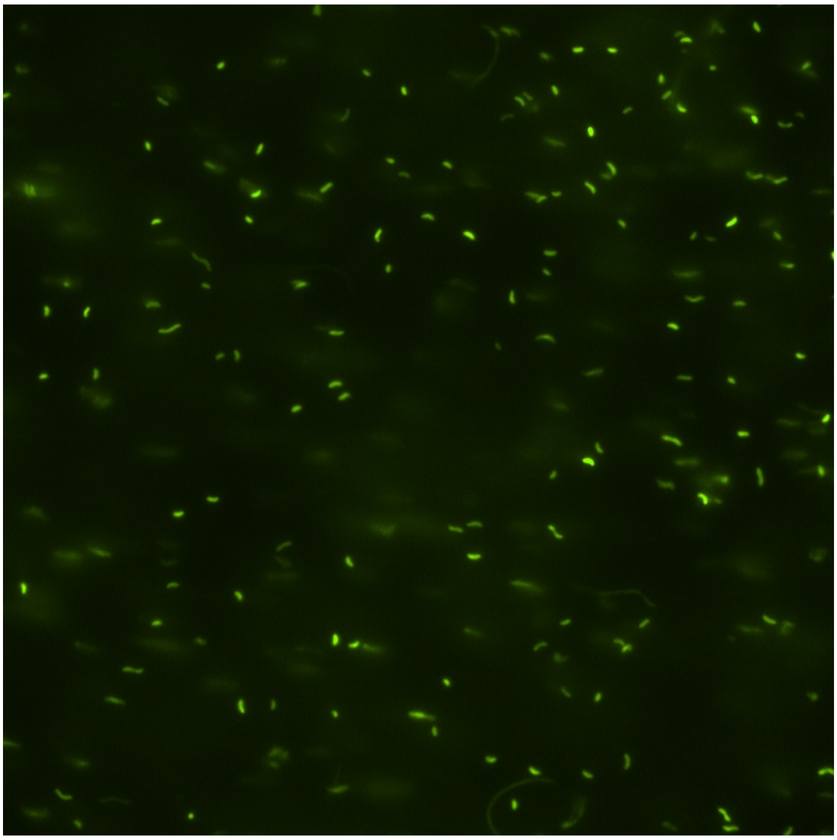

Supplementary Figure 5. Verification of *Vibrio cholerae* cell permeabilization. Permeabilization of cells prepared for single-cell RNA library construction was confirmed using Ovalbumin-Alexa Fluor 488 conjugate (Thermo Fisher Scientific). The intracellular accumulation of the green fluorescent signal indicates successful permeabilization of the bacterial cell envelope.

Supplementary Files

Supplementary File 1. Gene list showing significant differential gene expression (log_2FC) per cluster.

Supplementary Videos

Supplementary Video 1. Co-incubation of *V. cholerae* WT with *T. pyriformis* after 5 h in ASW. Real time video (phase contrast, transmitted light) showing the killing of *T. pyriformis* by *V. cholerae*. Video is representative of three independent experiments.

Supplementary Video 2. Co-incubation of *V. cholerae ΔmakA* with *T. pyriformis* after 5 h in ASW. Real time video (phase contrast, transmitted light) showing no killing of *T. pyriformis* by *V. cholerae ΔmakA*. Video is representative of three independent experiments.

Supplementary Video 3. Co-incubation of *V. cholerae ΔmakA-makA*$^+$ with *T. pyriformis* after 5 h in ASW. Real time video (phase contrast, transmitted light) showing that *makA* complementation *in trans* restores the killing of *T. pyriformis* by *V. cholerae*. Video is representative of three independent experiments.

Data Availability

The datasets presented in this study can be found online on NCBI under the accession code GSE307158.
